# Population-based validation of the RANO categories for extent of resection in glioblastoma

**DOI:** 10.1093/noajnl/vdag170

**Published:** 2026-06-30

**Authors:** Claes Johnstad, Ingerid Reinertsen, David Bouget, Okizeva Rapi, Asgeir S Jakola, Ole Solheim

**Affiliations:** Department of Neuromedicine and Movement Science, Faculty of Medicine and Health Sciences, Norwegian University of Science and Technology, Trondheim, Norway; Department of Health Research, SINTEF Digital, Trondheim, Norway; Department of Circulation and Medical Imaging, Faculty of Medicine and Health Sciences, Norwegian University of Science and Technology, Trondheim, Norway; Department of Health Research, SINTEF Digital, Trondheim, Norway; Section of Clinical Neuroscience, Institute of Neuroscience and Physiology, Sahlgrenska Academy, University of Gothenburg, Gothenburg, Sweden; Department of Neurosurgery, St. Olav’s Hospital, Trondheim University Hospital, Trondheim, Norway; Section of Clinical Neuroscience, Institute of Neuroscience and Physiology, Sahlgrenska Academy, University of Gothenburg, Gothenburg, Sweden; Department of Neurosurgery, Sahlgrenska University Hospital, Gothenburg, Sweden; Department of Neuromedicine and Movement Science, Faculty of Medicine and Health Sciences, Norwegian University of Science and Technology, Trondheim, Norway

**Keywords:** extent of resection, glioblastoma, overall survival, RANO resect

## Abstract

**Background:**

The RANO Resect group published the updated *RANO categories for extent of resection in glioblastoma* in 2023. Our aim was to provide a population-based validation of these categories.

**Methods:**

This population based retrospective cohort study included 470 consecutive patients treated at two centers in Norway and Sweden. None of the hospitals had a strategy to aim for supramarginal resections. Postoperative contrast-enhanced T1 and FLAIR volumes were used to group the patients according to the RANO categories. Cox models and Kaplan-Meier plots were used for survival analyses. We performed subgroup analyses of patients where the Stupp protocol was initiated, MGMT methylated tumors, and elderly patients (≥70 years).

**Results:**

Median overall survival was 16, 15, 11, and 6 months for the respective categories of supramaximal (Class 1), maximal (Class 2), submaximal resection (Class 3), and biopsy only (Class 4). Thirty-five patients (7.5%) were classified as Class 1, despite not aiming for resection beyond the contrast-enhancing tumor. This class had a median tumor volume of only 6 mL, smaller preoperative FLAIR volumes and was associated with improved survival compared to Class 2 (HR = 0.6, *P* < .0001) in the adjusted analysis. There was no significant difference between classes 3 and 4.

**Discussion and Conclusion:**

The RANO categories were associated with survival, but survival was shorter and differences across categories were less than in the original study. Maximal and supramaximal resections were associated with longer survival. One fourth of complete resections were incidentally classified as supramaximal resections, and these patients had a favorable prognosis.

Key Points

- The *RANO categories for EOR in glioblastoma* were associated with survival.
- The differences between classes were smaller than in the original study.
- One fourth of complete resections were incidentally categorized as *supramaximal*.

Importance of the Study

This is the first population-based validation of the RANO categories for extent of resection in glioblastoma that included all four classes. There was a clear association between the categories and overall survival. However, as compared to the original validation study, the differences between the classes were smaller, particularly regarding the benefits of supramaximal resection. Although this class was associated with better outcome, these supramaximal resections were achieved despite no established strategy of resection beyond the contrast-enhancing tumor; thus, they seem to represent a prognostically favorable patient group due to other factors. Additionally, the prognostic advantage of subtotal resection, compared to that of biopsy alone, was not significant. This was particularly true for elderly patients. Overall, the RANO categories may be useful for stratification in research, but their applicability in clinical practice remains limited.

Despite conflicting evidence from the few randomized controlled studies,^1^^,^^2^ an overwhelming body of observational studies concludes that resection improves survival in glioblastoma. Although making causal inference from heterogenous observational data is difficult, more homogeneous and reanalyzed trial data support the benefit of complete resections.^3^ Still, mainly complete or near-complete (gross total) resections have been found associated with a survival advantage.^4^ Between-hospital variations in biopsy frequencies exhibit no association to survival, supporting the limited benefit of partial resections over biopsies only in patients not suitable for gross total resections.^5^ However, the extent of resection threshold that improves survival has been discussed, without any clear consensus.^6-8^

Before the RANO resect group presented the *RANO categories for extent of resection (EOR) in glioblastoma*, there were no validated classifications for EOR and considerable variations in classification across studies.^9^ Karschnia et al developed a classification system based on the absolute postoperative residual tumor volume. They reported a biological gradient with large and stepwise survival differences between the four main categories—supramaximal resection, maximal resection, submaximal resection, and biopsy.^9^

RANO defined supramaximal resection (Class 1) as maximal resection with ≤5 mL of residual non-CE (contrast-enhancing) tumor volume, regardless of preoperative FLAIR-volume. It is known that the non-CE tumor volume contains tumor cells, although with a much lower density than in the CE part.^10^^,^^11^ Thus, the benefits of resection beyond the CE borders of the tumor have been frequently discussed in later years, with terms such as “FLAIR-ectomy” and “supramarginal/-total resection.” The benefits of such extended resections beyond the CE tumor volume have been unclear, but many observational studies suggest improved survival, either in general, or in subgroups of patients only,^9^^,^^12-18^ whereas some studies report no survival benefits.^19^^,^^20^ Some have reported a negative correlation between the preoperative non-CE tumor volume and survival,^21^ suggesting potential for confounding or selection bias in some FLAIR-ectomy studies.

Another controversy is the impact of resection on elderly patients. The ANOCEF trial found no difference in survival between surgical resection and biopsy only in patients of at least 70 years of age, contrasting observational studies that report a meaningful benefit from resection.^1^^,^^22^^,^^23^ A recent study by the RANO resect group shows survival benefits of maximal resection, but no difference in survival between supramaximal and maximal resection in elderly patients.^24^ Patient selection in the RANO studies have not been population-based. A Scandinavian, population-based validation study was published in 2023, but they did not include supramaximal resections (Class 1) or subgroup analysis of elderly patients.^25^

In this study, we sought to (1) validate the prognostic impact of the RANO classification in a population-based setting; (2) assess the incidence, characteristics, and prognosis of nonintended supramaximal resections; and (3) assess the prognostic impact of the RANO classification in the elderly (≥70 years).

## Methods

### Data

In this population-based study, we included 273 patients from St. Olavs Hospital in Norway and 197 patients from Sahlgrenska University Hospital, Sweden. Both centers serve exclusively defined geographical catchment areas, ensuring population-based referrals. All patients (age ≥18 years) with a histologically diagnosed glioblastoma and postoperative T1 and FLAIR scans from 2015 to 2024 in the Norwegian center and 2008-2016 in the Swedish center were included. All IDH-mutant tumors were excluded in line with the WHO 2021 classification of CNS tumors. During the study period, supramaximal resection was not an established surgical strategy at the two centers, and the CE tumor volume defined the surgical target. Patient-specific data on surgical adjuncts was not available for all patients. However, in the Norwegian center, motor mapping was regularly used in motor eloquent tumors, but awake resections were not performed. In the Swedish center, awake surgery was only performed exceptionally (<5% of cases), whereas motor mapping was used in <10% of cases. Overall survival was defined as the number of days from surgery to death.

### Measurements

**Figure 1.**
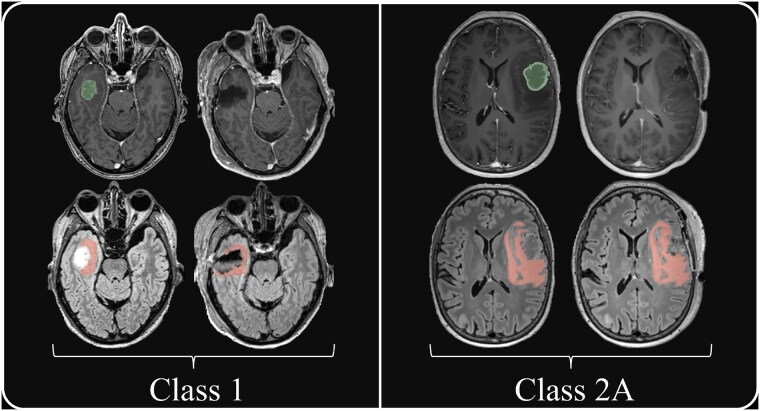
Illustration of two resections that were categorized as RANO Class 1 and Class 2A, respectively. The left column shows preoperative scans, and the right column shows postoperative scans. The upper row shows postgadolinium T1-weighted scans with segmentation of contrast-enhancing volume, and the lower row shows FLAIR scans with segmentation of peritumoral FLAIR volume. The preoperative scans were not used to categorize the resections but were included to show the differences in preoperative contrast-enhancing volume and peritumoral FLAIR volume.

Volumetric segmentations of the postgadolinium T1-weighted MRI scans were used to classify the patients into the respective four RANO classes and six subcategories for EOR in glioblastoma. All postoperative scans were performed within 48 hours after surgery; in most cases within 24 hours. Segmentations of CE tumor volumes were computed automatically using the validated software Raidionics and corrected manually.^26^ Postoperative FLAIR images of all patients in class 2A were manually segmented to define RANO Class 1. We used 3D Slicer version 5.8.0 for manual segmentation.^27^^,^^28^  Table 1 presents the definition of the respective RANO categories.^9^  Figure 1 shows an example of a Class 1 and a Class 2A resection. Tumor depth was defined as the shortest distance from the tumor center of mass to the brain surface. Multifocality was defined as two or more CE lesions separated by non-CE brain tissue.

**Table 1.** RANO categories for extent of resection in glioblastoma.

RANO category	Residual CE volume	Residual non-CE volume
Class 1: supramaximal resection	0 mL	≤ 5 mL
Class 2: maximal resection		
Class 2A: complete resection	0 mL	> 5 mL
Class 2B: near total resection	≤ 1 mL	
Class 3: submaximal resection		
Class 3A: subtotal resection	1 < x ≤ 5 mL	
Class 3B: partial resection	> 5 mL	
Class 4: Biopsy	Biopsy only	

Definition of the RANO categories for extent of resection in glioblastoma according to the original study by Karschnia et al.^1^ CE: contrast-enhancing.

### Treatment

Patients were grouped into five groups according to oncological treatment that was planned and initiated. Group A, Stupp protocol, was defined as patients who received 60 Gy radiation therapy and concomitant temozolomide, regardless of whether they completed the treatment protocol with adjuvant chemotherapy. Group B included all patients who received concomitant chemoradiotherapy with hypofractionated radiation (<60 Gy). Group C received radiotherapy plus only adjuvant chemotherapy. Group D received only adjuvant chemotherapy and no radiotherapy. Group E received neither chemotherapy nor radiotherapy.

### Statistics

All statistical analyses and illustrations were performed in RStudio, version 2025.05.1 + 513. Continuous variables were assessed for normality using Q-Q plots and Shapiro-Wilks test. Survival data were assessed with univariable and multivariable Cox proportional hazards regression models and Kaplan-Meier curves. Statistically significant variables from the univariable Cox models were included as covariables in the multivariable models. Due to the considerable differences in tumor volume between the categories seen in Table 2, CE tumor volume was also adjusted for. The two variables that violated the proportionality assumption of the Cox model—adjuvant treatment and, in the elderly subgroup, MGMT methylation status—were only used for stratification, and thus, effect estimates and *P* value were not presented. The data from the respective centers were clustered to account for inter-hospital differences; this would not affect the estimates but the confidence intervals. To enhance homogeneity of other treatments, all analyses were also performed in a subgroup of patients who underwent or were planned to undergo treatment according to the Stupp protocol.^29^ All analyses were also performed in an elderly subgroup of patients of at least 70 years of age and in a subgroup of patients with MGMT methylated tumors. Cases with missing values on any of the covariables were excluded from the multivariable Cox models. *P* value < 0.05 was considered statistically significant.

**Table 2.** Patient characteristics.

Variable	All patients (n = 470)	Class 1 (n = 35)	Class 2 (n = 183)	Class 3 (n = 125)	Class 4 (n = 127)	Stupp (n = 212)	MGMT+ (n = 203)	70+ (n = 142)
Age	65 (57-71)	66 (59-71)	63 (56-68)	64 (56-70)	68 (59-75)	60 (53-65)	65 (57-72)	74 (72-77)
CE volume	30 (9-53)	6 (3-14)	25 (8-45)	46 (24-66)	32 (10-49)	30 (9-56)	32 (10-56)	29 (14-48)
FLAIR volume	63 (28-114)	15 (6-26)	55 (32-102)	105 (66-166)	67 (36-104)	54 (27-121)	58 (29-115)	64 (28-102)
CE/FLAIR ratio	.43 (.25-.67)	.47 (.22-.92)	.37 (.22-.61)	.45 (.29-.66)	.44 (.23-.68)	.42 (.27-.70)	.42 (.22-.62)	.44 (.25-.67)
Postop. CE volume	.4 (.0-2.6)	.0 (.0-.0)	.0 (.0-.4)	3.5 (2.1-7.9)	Biopsy	.3 (.0-1.7)	.5 (.0-2.7)	.7 (.0-2.8)
Tumor depth (mm)	18 (13-27)	13 (10-18)	17 (11-25)	19 (15-25)	22 (16-32)	17 (11-23)	19 (13-27)	18 (13-27)
Overall survival	12 (6-19)	16 (13-23)	15 (10-24)	11 (7-19)	6 (3-13)	17 (12-26)	15 (8-24)	9 (5-14)
Sex								
Female	185 (39%)	13 (37%)	71 (39%)	50 (40%)	51 (40%)	76 (36%)	93 (46%)	54 (38%)
Male	284 (61%)	22 (63%)	111 (61%)	75 (60%)	76 (60%)	135 (64%)	110 (54%)	88 (62%)
KPS								
≥70	388 (83%)	34 (97%)	170 (93%)	105 (84%)	79 (62%)	193 (91%)	167 (82%)	112 (79%)
<70	78 (17%)	1 (3%)	12 (7%)	18 (14%)	47 (37%)	19 (9%)	34 (17%)	30 (21%)
Missing	4 (1%)	0 (0%)	1 (1%)	2 (2%)	1 (1%)	0 (0%)	2 (1%)	0 (0%)
Chemoradiotherapy								
A: Stupp	212 (45%)	18 (51%)	114 (62%)	63 (50%)	17 (13%)	212 (100%)	98 (48%)	11 (8%)
B: CCRT	78 (17%)	6 (17%)	17 (9%)	13 (10%)	42 (33%)	0	28 (14%)	51 (36%)
C: RT ± ACT	88 (19%)	7 (20%)	28 (15%)	24 (19%)	29 (23%)	0	27 (13%)	41 (29%)
D: ACT only	40 (9%)	2 (6%)	13 (7%)	12 (10%)	13 (10%)	0	31 (15%)	16 (11%)
E: No CRT	46 (10%)	2 (6%)	9 (5%)	10 (8%)	25 (20%)	0	16 (8%)	22 (15%)
Missing	6 (1%)	0 (0%)	2 (1%)	3 (2%)	1 (1%)	0	3 (2%)	1 (1%)
MGMT								
Methylated	203 (43%)	14 (40%)	87 (48%)	62 (50%)	40 (31%)	98 (46%)	203 (100%)	66 (46%)
Unmethylated	247 (53%)	20 (57%)	94 (51%)	60 (48%)	73 (57%)	106 (50%)	0 (0%)	70 (49%)
Missing	20 (4%)	1 (3%)	2 (1%)	3 (2%)	14 (11%)	8 (4%)	0 (0%)	6 (4%)
Multifocality								
Yes	98 (21%)	1 (3%)	25 (14%)	18 (14%)	54 (43%)	31 (15%)	45 (22%)	36 (25%)
No	371 (79%)	34 (97%)	158 (86%)	107 (86%)	73 (57%)	181 (85%)	158 (78%)	106 (75%)

Patient characteristics for the entire study cohort and for the respective patient subgroups and RANO categories for extent of resection. Median and interquartile range is reported for the continuous variables, whereas number and proportion of patients in each category are reported for the categorical variables. CE: contrast-enhancing; KPS: Karnofsky performance status; CCRT: concomitant chemoradiotherapy; RT: radiotherapy; ACT: adjuvant chemotherapy; CRT: chemoradiotherapy; MGMT: O_6_-methylguanine DNA-methyltransferase.

### Ethics

The study was performed as part of the project entitled “PREDICT: Decision support and artificial intelligence in neuro-oncology,” approval by the Regional Committees for Medical and Health Research Ethics (REK) Midt 2019/510 and the project entitled “Increased understanding of tumors involving the central nervous system tumors and their treatment,” approval by the Swedish Ethical Review Authority, Dnr 2022-02884-02.

## Results

In total, 470 consecutive patients with glioblastoma were included and analyzed, including 127 biopsies and 343 resections. Table 2 summarizes the characteristics of the sample population. The median age at the time of diagnosis was 65 years. The median overall survival was 12 months. Despite no established policy of supramaximal resection, 26.7% of the complete resections, and 7.5% of all patients, were classified as such (Class 1). Patients undergoing supramaximal resection had median preoperative tumor volume of 6 mL, compared to 30 mL for the total cohort. The preoperative FLAIR volume was also considerably smaller in this group (15 mL vs. 63 mL, n = 207). Median tumor depth increased with each resection category from Class 1 to Class 4. Two hundred twelve patients (45%) were started on Stupp protocol treatment. This subgroup had a median age of 60 years and a median survival of 17 months.

**Figure 2.**
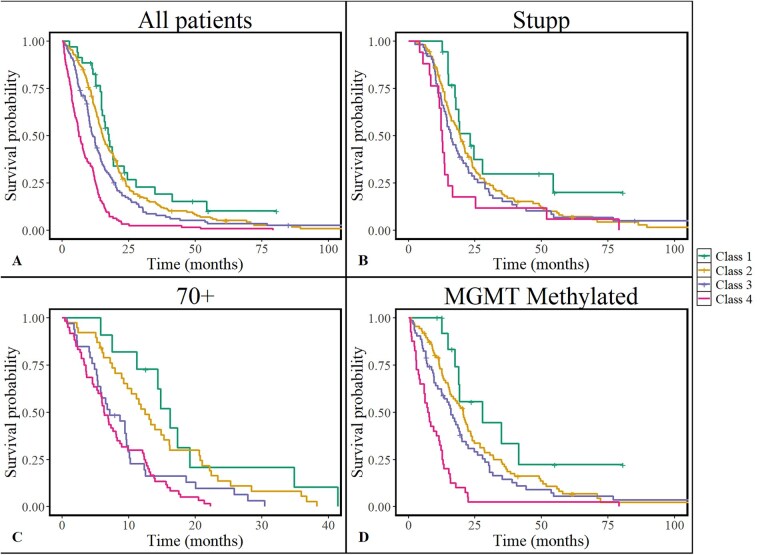
Kaplan-Meier curves for overall survival in all patients (A), patients started on treatment according to the Stupp protocol (B), elderly patients (C), and patients with MGMT methylated tumors (D). MGMT: O6-methylguanine DNA-methyltransferase.

Table 3 shows median overall survival with first and third quartiles for each RANO class and patient subgroup. Patients who underwent supramaximal resections (Class 1) had the longest median survival, with 16.2 months compared to 15.0 months in patients who underwent maximal resection (Class 2). In patients with MGMT methylated tumors or in patients started on Stupp protocol treatment, the differences in median survival between Class 1 and Class 2 were negligible. Similarly, there was only a twenty-five-day median survival difference between submaximal resections (Class 3) and biopsies (Class 4) in elderly patients. Otherwise, there was a stepwise decrease in median overall survival from Class 1 to Class 4. Figure 2 shows survival curves for each category. There is a clear difference in survival probability over time across the classes, but the gap between each class depends on which subpopulation is investigated.

**Table 3.** Median survival by subgroups.

	All	Class 1	Class 2	Class 3	Class 4
All	12.4 (6.3-19.4)	16.2 (12.7-23.4)	15.0 (10.0-23.5)	11.1 (6.6-19.0)	6.4 (3.3-12.6)
70+	8.5 (5.2-13.9)	14.8 (11.9-18.3)	12.2 (7.3-20.7)	7.2 (4.9-10.1)	6.4 (3.5-12.4)
MGMT Methylated	15.1 (7.9-24.4)	19.1 (15.2-33.1)	19.5 (11.4-31.3)	15.4 (7.0-25.8)	7.6 (3.0-12.8)
Stupp	17.2 (12.2-26.4)	19.1 (15.4-27.0)	19.2 (12.5-28.1)	16.0 (10.9-25.4)	12.8 (11.3-14.9)

Median overall survival for each RANO category by patient subgroups. First and third quartiles in parentheses.

**Figure 3.**
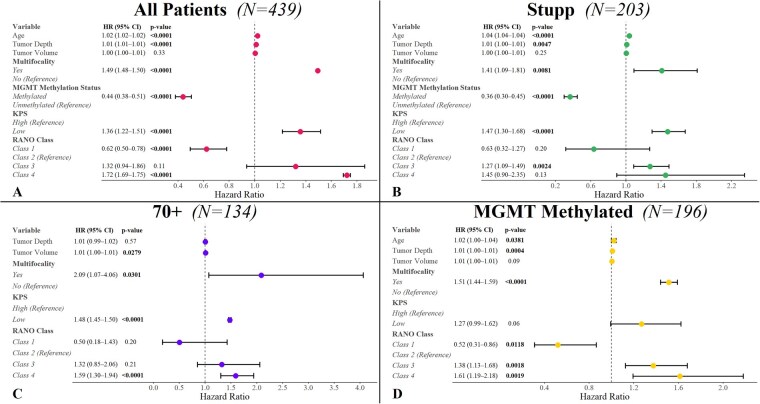
Forest plots of the multivariable Cox proportional hazards regression models for all patients (A), patients started on treatment according to the Stupp protocol (B), elderly patients (C), and patients with MGMT methylated tumors (D). HR: hazard ratio; CI: confidence interval; KPS: Karnofsky performance status; MGMT: O6-methylguanine DNA-methyltransferase.

Supplementary Table S2 shows the results of the univariable survival analyses. In addition to the RANO categories, age, treatment, KPS, multifocality, tumor depth, and MGMT status were statistically significant prognostic factors and were thus included as covariables in the multivariable Cox models. The results of the multivariable analyses are presented in Figure 3. With maximal CE resection (Class 2) as the reference value, supramaximal resection (Class 1) was associated with improved prognosis in unselected patients (HR = 0.62, *P* < .0001), whereas submaximal resection (Class 3) was not significantly different from maximal resection (HR = 1.32, *P* = .11), and biopsy only (Class 4) was associated with higher mortality risk (HR = 1.72, *P* < .0001). In patients who started the Stupp regimen, we observed shorter survival after submaximal resections (Class 3) (HR = 1.27, *P* = .0024), but no significant survival difference after supramaximal resections (HR = 0.63, *P* = .20) or biopsies (HR = 1.45, *P* = .13). In the elderly subgroup, only the biopsy class had significantly different and worse survival from that of the maximal resection class (HR = 1.59, *P* < .0001). In patients with MGMT methylated tumors, the survival differences between the classes were comparable to those of the full-data analyses, but the difference between maximal resection (Class 2) and submaximal resection (Class 3) was also significant in this subgroup (HR = 1.38, *P* = .0018).

Supplementary Figure S1 displays pairwise Cox analyses with HR between each of the RANO classes. These tile plots show progressively increasing mortality risks from Class 1 to Class 4. The same trends were seen in the subgroup analyses, but the observed associations had lower statistical significance. There was no statistically significant difference between submaximal resection (Class 3) and biopsy alone (Class 4). The pairwise HRs for this comparison, with corresponding confidence intervals, were 0.77 (0.54-1.10), 0.88 (0.63-1.22), 0.83 (0.44-1.58), and 0.85 (0.52-1.41) for the total cohort, Stupp subgroup, elderly subgroup, and MGMT methylated subgroup, respectively.

## Discussion

In this retrospective, population-based observational study of 470 patients, we validated the prognostic value of the RANO categories for extent of resection in glioblastoma. The prognostic value was largely statistically confirmed, but the survival differences across resection classes were considerably less in this cohort than in the original RANO study. For stratification or adjustment purposes in, for example, clinical trials, the RANO classification works well. Still, clinicians should be cautious to make causal inference from the retrospective studies on the RANO categories. In this study, only supramaximal (Class 1) and maximal resections (Class 2) were associated with better prognoses compared to biopsy only, supporting that limited debulking may not be much better than biopsy alone.

While the RANO Resect group found median survival of 29, 19, 16, and 10 months for the respective resection classes,^9^ suggesting median survival difference of 10 months between supramaximal and maximal resections, the median survival times in our study were 16, 15, 11, and 6 months, respectively. The discrepancies in the results could be explained by the differences in the study populations. The RANO study included only on average seven patients per center per year from seven high volume tertiary referral centers from 2003 to 2022.^9^ The respective cohorts had median overall survival ranging from 12 to 32 months, and only 7% underwent biopsy only,^9^ in contrast to the 21% in a similar population-based study.^25^ These figures suggest a possible selection bias and therefore limited external validity.

Like in the RANO resect study,^9^ patients who underwent supramaximal resection (Class 1), exhibited the best prognoses. However, the absolute survival difference was only 1 month in unselected patients, compared to ten in the original validation.^9^ Although supramaximal resection (Class 1) was significantly associated with lower mortality risk, the clinical significance of 1 month difference in median survival may be questionable. Also, the patients in Class 1 harbored much smaller tumors—regarding both CE volume and FLAIR volume—than the other resection categories, suggesting a potential for confounding. Further, in the subgroup analyses of patients treated with the Stupp protocol or in elderly patients, there was no significant advantage of supramaximal resection (Class 1) over maximal resection (Class 2), suggesting that the observed association could be influenced by confounding by adjuvant treatment- or age-related factors. In the original RANO publication, 76 (21%) of the complete resections were Class 1.^9^ Other validation studies also found superior outcome in this category, with supramaximal-to-complete resection ratios of 57%^30^ and 63%,^31^ respectively. In this study, 27% of complete resections (Classes 1 and 2A) were defined as supramaximal resections (Class 1), despite no intention of resection beyond the CE volume at the two centers. These figures may indicate that the RANO definition of supramaximal resection may not only catch intended FLAIR-ectomies, but also approximately one fourth of the maximal resections, typically of superficial tumors with small preoperative FLAIR and/or CE volumes. Therefore, Class 1 may not always be, surgically speaking, supramaximal. Also noteworthily, the volume of the non-CE tumor on postoperative MRI will vary considerably with the timing of the scan, use of corticosteroids, MGMT methylation status,^32^^,^^33^ volume,^34^ and location of the tumor,^35^^,^^36^ indicating the considerable potential for confounding factors. Thus, it remains unknown whether undergoing supramaximal surgery provides additional survival benefits, or if Class 1 is more often obtained in a group of patients with better prognosis due to other underlying factors.

Maximal resection (Class 2) was also associated with longer survival, with a significantly lower mortality risk compared to the biopsy class (Class 4). Although observed median survival in this class was longer than that of submaximal resections (Class 3) as well, the difference was not statistically significant in unselected patients. However, in patients where treatment with the Stupp protocol was initiated, the statistical analyses showed a significant advantage of maximal resection (Class 2) over submaximal resection (Class 3), supporting total or near-total resection in patients that are eligible to receive the most aggressive postoperative treatment. A previous population-based study supports the findings of superior outcome after maximal resections compared to biopsy alone,^25^ but studies comparing maximal with submaximal resections found no significant differences.^30^^,^^31^ In this study, there was no significant survival difference between submaximal resection (Class 3) and biopsy only (Class 4). Most previous studies did not include the biopsy class, but one study showed overall survival of 9 months and 6 months in the submaximal and biopsy classes, respectively,^25^ even smaller differences than in this study. A meta-analysis of the aforementioned validation studies of the RANO categories concluded that supramaximal resection (Class 1) was associated with longer overall survival, but regarding the other resection classes, only complete CE resection (Class 2A) was significantly different from the subtotal resection and biopsy groups.^37^ These results could support an “all-or-nothing” strategy in glioblastoma surgery, aiming for either complete/near-complete resection or biopsy alone. However, prospective clinical trials are needed to evaluate the independent survival benefits of the surgical approach.

In elderly patients, we found no significant survival benefit from supramaximal resection (Class 1) compared to maximal resection (Class 2), but maximal resection was associated with superior outcome compared to biopsy alone (Class 4), in line with a recent study by the RANO Resect group.^24^ In this study on elderly patients, they also found attenuation of the benefit of maximal resection with increasing age, with no benefit from 80 years of age, supporting a more conservative approach in the eldest segment of the population. Furthermore, this study showed no significant differences in survival between the submaximal resections (Class 3) and the biopsies (Class 4). These findings indicate sparse benefits of resection, particularly submaximal resection, in elderly patients with glioblastoma, thus supporting previous studies.^1^^,^^2^^,^^38^ Although these studies have shown differences in overall survival between those who underwent resection and those who underwent biopsy, the benefits are considerably less than for younger patients. Hence, age is an important factor to keep in mind when considering surgical resection versus biopsy alone and when using the RANO categories for stratification in clinical studies. However, causal inference cannot be made from this retrospective analysis alone. Seeing the differences in tumor volume, KPS, and postoperative treatment, the RANO categories evidently stratify the patients by many factors besides EOR, and these potential confounders could also influence the lack of observed differences between submaximal resection (Class 3) and biopsy alone (Class 4). There is also potential for confounding by comorbidity, frailty, symptom burden, subependymal growth, and other factors that may affect clinical decision making. Most likely, many such confounders may prognostically act in favor of resections over biopsy only. Even so, the confidence intervals were relatively wide, and the sample sizes of the subgroup analyses were considerably smaller than that of the full cohort. Hence, a lack of statistical power could also have contributed to these findings.

The main strength of this study is the population-based cohort. The two centers treat all glioblastoma patients within their geographical catchment region, thus minimizing selection bias and ensuring a cohort with baseline characteristics comparable to the general glioblastoma population.^39^^,^^40^ The study is limited by its retrospective design and possibly limited statistical power for some subgroup analyses. Particularly, the supramaximal resection class and the biopsy class had sparse sample sizes in the Stupp subgroup analyses, whereas the elderly subgroup mainly lacked data on supramaximal resections. The retrospective data were highly confounded across RANO classes, making it difficult to draw any inference on the therapeutic benefits of various extents of resection. Hence, this study was limited to validation of these categories as a prognostic stratification tool. Furthermore, the data were collected over the course of 17 years, potentially introducing some temporal heterogeneity. Although adjuvant oncological treatment of glioblastoma has not changed much during this period, other factors—such as availability of molecular markers, change in imaging protocols, and smaller changes in surgical adjuncts—may have influenced the data. There may also have been differences between the two centers included. Although clustering by hospital was performed in the multivariable models to account for variance, it would not eliminate bias. Hence, center-specific analyses were performed and presented in Supplementary Table S3.

## Conclusion

In this population-based validation study, the prognostic value of the RANO categories for extent of resection in glioblastoma was largely confirmed. However, overall survival was shorter and differences across resection classes were considerably smaller than previously reported. Class 1 resections are sometimes achieved even without FLAIR volume as surgical target, especially in small tumors. It remains unknown whether undergoing supramaximal surgery provides additional survival benefits, or if Class 1 merely represents a group of patients with better prognosis due to other underlying factors. The RANO classes may serve as a prognostic stratification tool in future research, but their clinical applicability remains limited.

## Supplementary Material

vdag170_Supplementary_Data

## Data Availability

The datasets presented in this article are not readily available because of restricted access by the General Data Protection Regulation (GDPR). Requests to access the datasets should be directed to Claes.Johnstad@ntnu.no.

## Data Collection

All authors.

## Approval of Manuscript

All authors.

## Previous Presentations

Presented on October 23, 2025, at the Annual Meeting of the Norwegian Neurosurgical Association in Oslo, Norway, and on April 10, 2026, at SNOG Northern Lights Neuroscience Symposium in Copenhagen, Denmark.

## References

[1] Laigle-Donadey F , Metellus P, Guyotat J, et al Surgery for glioblastomas in the elderly: an association des neuro-oncologues d’Expression française (ANOCEF) trial. J Neurosurg. 2023;138:1199-1205. 10.3171/2022.8.jns221068 36242578

[2] Vuorinen V , Hinkka S, Färkkilä M, Jääskeläinen J. Debulking or biopsy of malignant glioma in elderly people—a randomised study. Acta Neurochir (Wien). 2003;145:5-10. 10.1007/s00701-002-1030-6 12545256

[3] Stummer W , Reulen H-J, Meinel T, ALA-Glioma Study Group, et al Extent of resection and survival in glioblastoma multiforme: identification of and adjustment for bias. Neurosurgery. 2008;62:564-576. 10.1227/01.neu.0000317304.31579.17 18425006

[4] Brown TJ , Brennan MC, Li M, et al Association of the extent of resection with survival in glioblastoma. JAMA Oncol. 2016;2:1460-1469. 10.1001/jamaoncol.2016.1373 27310651 PMC6438173

[5] Viozzi I , Hannink G, Ardon H, et al Between-hospital variation in biopsy indication for patients with newly diagnosed glioblastoma in the Dutch quality registry for neurosurgery. J Neurooncol. 2025;172:625-632. 10.1007/s11060-025-04959-5 39913047 PMC11968504

[6] Sanai N , Polley M-Y, McDermott MW, Parsa AT, Berger MS. An extent of resection threshold for newly diagnosed glioblastomas. J Neurosurg. 2011;115:3-8. 10.3171/2011.2.jns10998 21417701

[7] Lacroix M , Abi-Said D, Fourney DR, et al A multivariate analysis of 416 patients with glioblastoma multiforme: prognosis, extent of resection, and survival. J Neurosurg. 2001;95:190-198. 10.3171/jns.2001.95.2.0190 11780887

[8] Chaichana KL , Cabrera-Aldana EE, Jusue-Torres I, et al When gross total resection of a glioblastoma is possible, how much resection should Be achieved? World Neurosurg. 2014/07/01/ 2014;82:e257-e265. 10.1016/j.wneu.2014.01.019 24508595

[9] Karschnia P , Young JS, Dono A, et al Prognostic validation of a new classification system for extent of resection in glioblastoma: a report of the RANO *resect* group. Neuro Oncol. 2023;25:940-954. 10.1093/neuonc/noac193 35961053 PMC10158281

[10] Barajas RF Jr. , Phillips JJ, Parvataneni R, et al Regional variation in histopathologic features of tumor specimens from treatment-naive glioblastoma correlates with anatomic and physiologic MR imaging. Neuro Oncol. 2012;14:942-954. 10.1093/neuonc/nos128 22711606 PMC3379808

[11] Gill BJ , Pisapia DJ, Malone HR, et al MRI-localized biopsies reveal subtype-specific differences in molecular and cellular composition at the margins of glioblastoma. Proceedings of the National Academy of Sciences. 2014;111:12550-12555. 10.1073/pnas.1405839111 PMC4151734 25114226

[12] Roh TH , Kang S-G, Moon JH, et al Survival benefit of lobectomy over gross-total resection without lobectomy in cases of glioblastoma in the noneloquent area: a retrospective study. J Neurosurg. 2020;132:895-901. 10.3171/2018.12.jns182558 30835701

[13] Yoo J , Yoon S-J, Kim KH, et al Patterns of recurrence according to the extent of resection in patients with IDH–wild-type glioblastoma: a retrospective study. J Neurosurg. 2022;137:533-543. 10.3171/2021.10.jns211491 34972087

[14] Molinaro AM , Hervey-Jumper S, Morshed RA, et al Association of maximal extent of resection of contrast-enhanced and non-contrast-enhanced tumor with survival within molecular subgroups of patients with newly diagnosed glioblastoma. JAMA Oncol. 2020;6:495-503. 10.1001/jamaoncol.2019.6143 32027343 PMC7042822

[15] Glenn CA , Baker CM, Conner AK, et al An examination of the role of supramaximal resection of temporal lobe glioblastoma multiforme. World Neurosurg/ 2018;114:e747-e755. 10.1016/j.wneu.2018.03.072 29555603

[16] Pessina F , Navarria P, Cozzi L, et al Maximize surgical resection beyond contrast-enhancing boundaries in newly diagnosed glioblastoma multiforme: is it useful and safe? A single institution retrospective experience. J Neurooncol. 2017;135:129-139. 10.1007/s11060-017-2559-9 28689368

[17] Di L , Shah AH, Mahavadi A, et al Radical supramaximal resection for newly diagnosed left-sided eloquent glioblastoma: safety and improved survival over gross-total resection. J Neurosurg. 2023;138:62-69. 10.3171/2022.3.jns212399 35623362

[18] Vivas-Buitrago T , Domingo RA, Tripathi S, et al Influence of supramarginal resection on survival outcomes after gross-total resection of IDH–wild-type glioblastoma. J Neurosurg. 2022;136:1-8. 10.3171/2020.10.jns203366 PMC9248270 34087795

[19] Mampre D , Ehresman J, Pinilla-Monsalve G, et al Extending the resection beyond the contrast-enhancement for glioblastoma: feasibility, efficacy, and outcomes. Br J Neurosurg. 2018;32:528-535. 10.1080/02688697.2018.1498450 30073866

[20] Alafandi A , Van Garderen KA, Klein S, et al Association of pre-radiotherapy tumour burden and overall survival in newly diagnosed glioblastoma adjusted for MGMT promoter methylation status. Eur J Cancer. 2023;188:122-130. 10.1016/j.ejca.2023.04.021 37235895

[21] Schoenegger K , Oberndorfer S, Wuschitz B, et al Peritumoral edema on MRI at initial diagnosis: an independent prognostic factor for glioblastoma? Eur J Neurol. 2009;16:874-878. 10.1111/j.1468-1331.2009.02613.x 19473360

[22] Lopez-Rivera V , Dono A, Lewis CT, et al Extent of resection and survival outcomes of geriatric patients with glioblastoma: is there benefit from aggressive surgery? Clin Neurol Neurosurg. Mar 2021;202:106474. 10.1016/j.clineuro.2021.106474 33454497

[23] Cunha MLVD , Esmeraldo ACS, Henriques LAW, Santos MAMD Jr, Medeiros RTR, Botelho RV. Elderly patients with glioblastoma: the impact of surgical resection extent on survival. Rev Assoc Med Bras. 2019;65:937-945. 10.1590/1806-9282.65.7.937 31389501

[24] Teske N , Dono A, Young JS, et al Associations of supramaximal resection with outcome in glioblastoma across age groups: a report of the RANO resect group. Neuro Oncol. 2026;28:470-484. 10.1093/neuonc/noaf239 41137668 PMC12979034

[25] Bjorland LS , Mahesparan R, Fluge Ø, Gilje B, Dæhli Kurz K, Farbu E. Impact of extent of resection on outcome from glioblastoma using the RANO resect group classification system: a retrospective, population-based cohort study. Neurooncol Adv. 2023;5:vdad126. 10.1093/noajnl/vdad126 37868696 PMC10590175

[26] Majewska P , Holden Helland R, Ferles A, et al Prognostic value of manual versus automatic methods for assessing extents of resection and residual tumor volume in glioblastoma. J Neurosurg. 2025;142:1298-1306. 10.3171/2024.8.jns24415 39823581

[27] Fedorov A , Beichel R, Kalpathy-Cramer J, et al 3D slicer as an image computing platform for the quantitative imaging network. Magn Reson Imaging. 2012;30:1323-1341. 10.1016/j.mri.2012.05.001 22770690 PMC3466397

[28] Slicer D. 3D Slicer image computing platform. Updated 2024-02-19. https://www.slicer.org/

[29] Stupp R , Mason WP, Van Den Bent MJ, National Cancer Institute of Canada Clinical Trials Group, et al Radiotherapy plus concomitant and adjuvant temozolomide for glioblastoma. N Engl J Med. 2005;352:987-996. 10.1056/nejmoa043330 15758009

[30] Tropeano MP , Raspagliesi L, Bono BC, et al Supramaximal resection: retrospective study on IDH-wildtype glioblastomas based on the new RANO-resect classification. Acta Neurochir (Wien). 2024;166:196. 10.1007/s00701-024-06090-2 38676720

[31] Park YW , Choi KS, Foltyn-Dumitru M, et al Incorporating supramaximal resection into survival stratification of IDH-wildtype glioblastoma: a refined multi-institutional recursive partitioning analysis. Clin Cancer Res. 2024;30:4866-4875. 10.1158/1078-0432.Ccr-23-3845 38829906 PMC12125701

[32] Carrillo JA , Lai A, Nghiemphu PL, et al Relationship between tumor enhancement, edema, IDH1 mutational status, MGMT promoter methylation, and survival in glioblastoma. AJNR Am J Neuroradiol. 2012;33:1349-1355. 10.3174/ajnr.A2950 22322613 PMC7965490

[33] Suh CH , Kim HS, Jung SC, Choi CG, Kim SJ. Clinically relevant imaging features for *MGMT* promoter methylation in multiple glioblastoma studies: a systematic review and meta-analysis. American Journal of Neuroradiology. 2018;39:1439-1445. 10.3174/ajnr.A5711 30002055 PMC7410549

[34] Henker C , Hiepel MC, Kriesen T, et al Volumetric assessment of ­glioblastoma and its predictive value for survival. Acta Neurochir (Wien). 2019;161:1723-1732. 10.1007/s00701-019-03966-6 31254065

[35] Zhang M , Ye F, Su M, Cui M, Chen H, Ma X. The prognostic role of peritumoral edema in patients with newly diagnosed glioblastoma: a retrospective analysis. J Clin Neurosci. 2021;89:249-257. 10.1016/j.jocn.2021.04.042 34119276

[36] Seidel C , Dörner N, Osswald M, et al Does age matter? - a MRI study on peritumoral edema in newly diagnosed primary glioblastoma. BMC Cancer. 2011;11:127. 10.1186/1471-2407-11-127 21481277 PMC3094323

[37] Wach J , Vychopen M, Güresir E. Prognostic revalidation of RANO categories for extent of resection in glioblastoma: a reconstruction of individual patient data. J Neurooncol. 2025;172:515-525. 10.1007/s11060-025-04950-0 39992571 PMC11968501

[38] Bandopadhay J , Pichardo-Rojas PS, Cho R, et al Assessment of the RANO-resect criteria in elderly patients with glioblastoma. J Neurooncol. 2025;175:721-729. 10.1007/s11060-025-05168-w 40742500

[39] Pham J , Cote DJ, Kang K, et al Treatment practices and survival outcomes for IDH-wildtype glioblastoma patients according to MGMT promoter methylation status: insights from the U.S. National cancer database. J Neurooncol. 2025;172:655-665. 10.1007/s11060-025-04952-y 39907975 PMC11968476

[40] Price M , Ballard C, Benedetti J, et al CBTRUS statistical report: primary brain and other central nervous system tumors diagnosed in the United States in 2017–2021. Neuro Oncol. 2024;26:vi1-vi85. 10.1093/neuonc/noae145 39371035 PMC11456825

