# Supplementary material for: Population-based validation of the RANO categories for extent of resection in glioblastoma
Source: Neurooncol Adv. 2026 Jun 30;8(1):vdag170. doi: 10.1093/noajnl/vdag170 (PMC13395087; doi:10.1093/noajnl/vdag170)
Supplement: vdag170_Supplementary_Data [file vdag170_supplementary_data.docx]

| Table S1: Median Overall Survival by Center (Norway \| Sweden) | | | | | | | | | | |
| --- | --- | --- | --- | --- | --- | --- | --- | --- | --- | --- |
| Subgroup | Overall | | Class 1 | | Class 2 | | Class 3 | | Class 4 | |
| All patients | 11.5 | 13.1 | 16.3 | 15.6 | 13.7 | 15.8 | 12.9 | 10.5 | 6.0 | 9.4 |
| Stupp | 14.9 | 19.0 | 23.3 | 15.1 | 15.6 | 20.3 | 14.5 | 18.2 | 12.8 | 11.7 |
| 70+ | 7.8 | 9.7 | 11.9 | 19.2 | 13.4 | 11.6 | 7.9 | 7.0 | 6.4 | 9.0 |
| MGMT methylated | 13.6 | 15.7 | 19.3 | 17.5 | 20.4 | 19.1 | 16.0 | 13.5 | 6.6 | 10.5 |

*Table S1: Center-specific median overall survival.*

| Table S2: Results of the Cox Proportional Hazards Regression Models | | | | | |
| --- | --- | --- | --- | --- | --- |
| Variable (n=uni\|multi) | *Univariable* | | | *Multivariable* | |
|  | *HR (CI)* | | *p* | *HR (CI)* | *p* |
| All Patients (N_multivariable_ = 439) | | | | | |
| Age | 1.03 (1.03-1.04) | **<0.0001** | | 1.02 (1.02-1.02) | **<0.0001** |
| Tumor Volume | 1.00 (1.00-1.01) | 0.13 | | 1.00 (1.00-1.01) | 0.33 |
| Tumor Depth | 1.01 (1.01-1.02) | **0.0005** | | 1.01 (1.01-1.01) | **<0.0001** |
| Multifocality |  |  | |  |  |
| Yes | 1.84 (1.46-2.30) | **<0.0001** | | 1.49 (1.48-1.50) | **<0.0001** |
| No | Reference | | | Reference | |
| Preoperative KPS |  |  | |  |  |
| ≥ 70 | Reference | | | Reference | |
| < 70 | 1.81 (1.41-2.32) | **<0.0001** | | 1.36 (1.22-1.51) | **<0.0001** |
| RANO categories |  |  | |  |  |
| Class 1 (n=35\|34) | 0.77 (0.51-1.15) | 0.20 | | 0.62 (0.50-0.78) | **<0.0001** |
| Class 2 (n=183\|178) | Reference | | | Reference | |
| Class 3 (n=125\|118) | 1.33 (1.05-1.68) | **0.02** | | 1.32 (0.94-1.86) | 0.11 |
| Class 4 (n=127\|109) | 2.73 (2.16-3.46) | **<0.0001** | | 1.72 (1.69-1.75) | **<0.0001** |
| MGMT status |  |  | |  |  |
| Methylated | 0.53 (0.43-0.65) | **<0.0001** | | 0.44 (0.38-0.51) | **<0.0001** |
| Unmethylated | Reference | | | Reference | |
| Treatment | Used for stratification only | | | | |
| Stupp Subgroup (N_multivariable_ = 203) | | | | | |
| Age | 1.04 (1.02-1.05) | | **<0.0001** | 1.04 (1.04-1.04) | **<0.0001** |
| Tumor Depth | 1.01 (1.00-1.02) | | 0.17 | 1.01 (1.00-1.01) | **0.0048** |
| Tumor Volume | 1.00 (1.00-1.01) | | 0.040 | 1.00 (1.00-1.01) | 0.25 |
| Multifocality |  | |  |  |  |
| Yes | 1.35 (0.91-2.01) | | 0.13 | 1.41 (1.09-1.81) | **0.0081** |
| No | Reference | | | Reference | |
| Preoperative KPS |  | |  |  |  |
| ≥ 70 | Reference | | | Reference | |
| < 70 | 1.12 (0.68-1.84) | | 0.66 | 1.47 (1.30-1.68) | **<0.0001** |
| RANO categories |  | |  |  |  |
| Class 1 (n=18\|17) | 0.63 (0.35-1.14) | | 0.13 | 0.63 (0.32-1.68) | 0.20 |
| Class 2 (n=114\|111) | Reference | | | Reference | |
| Class 3 (n=63\|60) | 1.14 (0.83-1.57) | | 0.42 | 1.27 (1.09-1.49) | **0.0024** |
| Class 4 (n=17\|15) | 1.75 (1.05-2.94) | | **0.033** | 1.45 (0.90-2.35) | 0.13 |
| MGMT status |  | |  |  |  |
| Methylated | 0.44 (0.33-0.59) | | **<0.0001** | 0.36 (0.30-0.45) | **<0.0001** |
| Unmethylated | Reference | | | Reference | |
| 70+ Subgroup (N_multivariable_ = 134) | | | | | |
| Age | 1.01 (0.97-1.06) | | 0.60 |  |  |
| Tumor Depth | 1.01 (1.00-1.03) | | 0.14 | 1.01 (0.99-1.02) | 0.57 |
| Tumor Volume | 1.00 (1.00-1.01)) | | 0.31 | 1.01 (1.00-1.01) | **0.028** |
| Multifocality |  | |  |  |  |
| Yes | 1.99 (1.35-2.95) | | **0.0006** | 2.09 (1.07-4.06) | **0.030** |
| No | Reference | | | Reference | |
| Preoperative KPS |  | |  |  |  |
| ≥ 70 | Reference | | | Reference | |
| < 70 | 2.12 (1.41-3.20) | | **0.0003** | 1.48 (1.45-1.50) | **<0.0001** |
| RANO categories |  | |  |  |  |
| Class 1 (n=11\|11) | 0.66 (0.32-1.37) | | 0.26 | 0.50 (0.18-1.43) | 0.20 |
| Class 2 (n=38\|38) | Reference | | | Reference | |
| Class 3 (n=33\|30) | 1.83 (1.13-2.98) | | 0.014 | 1.32 (0.85-2.06) | 0.21 |
| Class 4 (n=60\|55) | 2.35 (1.53-3.63) | | **0.0001** | 1.59 (1.30-1.94) | **<0.0001** |
| MGMT status | Used for stratification only | | | | |
| Treatment | Used for stratification only | | | | |
| MGMT Methylated Subgroup (N_multivariable_ = 196) | | | | | |
| Age | 1.04 (1.02-1.05) | | **<0.0001** | 1.02 (1.00-1.04) | **0.038** |
| Tumor Depth | 1.02 (1.00-1.03) | | **0.013** | 1.01 (1.00-1.01) | **0.0004** |
| Tumor Volume | 1.00 (1.00-1.01) | | **0.040** | 1.01 (1.00-1.01) | 0.086 |
| Multifocality |  | |  |  |  |
| Yes | 1.92 (1.36-2.72) | | **0.0002** | 1.51 (1.44-1.59) | **<0.0001** |
| No | Reference | | | Reference | |
| Preoperative KPS |  | |  |  |  |
| ≥ 70 | Reference | | | Reference | |
| < 70 | 1.71 (1.16-2.51) | | **0.0064** | 1.27 (1.00-1.62) | 0.055 |
| RANO categories |  | |  |  |  |
| Class 1 (n=14\|14) | 0.56 (0.27-1.15) | | 0.11 | 0.52 (0.31-0.87) | **0.012** |
| Class 2 (n=87\|84) | Reference | | | Reference | |
| Class 3 (n=62\|61) | 1.26 (0.89-1.77) | | 0.19 | 1.38 (1.13-1.68) | **0.0018** |
| Class 4 (n=40\|37) | 3.05 (2.05-4.52) | | **<0.0001** | 1.61 (1.19-2.18) | **0.0019** |
| Treatment | Used for stratification only | | | | |

Table S2: Results of the Cox proportional hazards models. HR: hazard ratio; CI: confidence interval; KPS: Karnofsky performance status; MGMT: O_6_-methylguanine DNA-methyltransferase.

| Table S3: Center-Specific Results of the Multivariable Cox Models | | | | | |
| --- | --- | --- | --- | --- | --- |
| Variable | *Norway* | | | *Sweden* | |
|  | *HR (CI)* | | *p* | *HR (CI)* | *p* |
| All Patients | | | | | |
| Age | 1.02 (1.01-1.04) | **0.0019** | | 1.02 (1.00-1.04) | 0.070 |
| Tumor Volume | 1.00 (0.99-1.00) | 0.79 | | 1.01 (1.00-1.02) | **0.0048** |
| Tumor Depth | 1.01 (1.00-1.02) | 0.12 | | 1.01 (1.00-1.02) | 0.24 |
| Multifocality |  |  | |  |  |
| Yes | 1.51 (1.05-2.18) | **0.028** | | 1.59 (1.03-2.47) | **0.038** |
| No | Reference | | | Reference | |
| Preoperative KPS |  |  | |  |  |
| ≥ 70 | Reference | | | Reference | |
| < 70 | 1.44 (1.03-2.01) | **0.035** | | 1.30 (0.70-2.41) | 0.41 |
| RANO categories |  |  | |  |  |
| Class 1 (n=22\|12) | 0.52 (0.29-0.95) | **0.033** | | 0.68 (0.34-1.37) | 0.28 |
| Class 2 (n=86\|92) | Reference | | | Reference | |
| Class 3 (n=58\|60) | 1.02 (0.70-1.48) | 0.91 | | 1.71 (1.19-2.46) | **0.0037** |
| Class 4 (n=94\|15) | 1.63 (1.11-2.41) | **0.014** | | 1.23 (0.63-2.39) | 0.55 |
| MGMT status |  |  | |  |  |
| Methylated | 0.46 (0.34-0.62) | **<0.0001** | | 0.34 (0.23-0.50) | **<0.0001** |
| Unmethylated | Reference | | | Reference | |
| Treatment | Used for stratification only | | | | |
| Stupp Subgroup | | | | | |
| Age | 1.04 (1.01-1.06) | | **0.0040** | 1.05 (1.02-1.08) | **0.0003** |
| Tumor Depth | 1.00 (0.98-1.02) | | 0.94 | 1.01 (0.99-1.03) | 0.54 |
| Tumor Volume | 1.00 (0.99-1.01) | | 0.83 | 1.01 (1.00-1.02) | 0.077 |
| Multifocality |  | |  |  |  |
| Yes | 1.88 (0.97-3.64) | | 0.062 | 1.44 (0.75-2.79) | 0.27 |
| No | Reference | | | Reference | |
| Preoperative KPS |  | |  |  |  |
| ≥ 70 | Reference | | | Reference | |
| < 70 | 1.32 (0.61-2.83) | | 0.48 | 1.75 (0.75-4.08) | 0.19 |
| RANO categories |  | |  |  |  |
| Class 1 (n=12\|5) | 0.42 (0.17-1.00) | | 0.051 | 1.33 (0.51-3.48) | 0.56 |
| Class 2 (n=55\|56) | Reference | | | Reference | |
| Class 3 (n=29\|31) | 1.21 (0.72-2.05) | | 0.48 | 1.49 (0.91-2.43) | 0.11 |
| Class 4 (n=13\|2) | 1.08 (0.52-2.25) | | 0.84 | 8.46 (1.62-44.2) | **0.011** |
| MGMT status |  | |  |  |  |
| Methylated | 0.41 (0.26-0.65) | | **0.0001** | 0.28 (0.16-0.48) | **<0.0001** |
| Unmethylated | Reference | | | Reference | |
| 70+ Subgroup | | | | | |
| Tumor Depth | 1.02 (0.99-1.05) | | 0.12 | 0.99 (0.96-1.02) | 0.58 |
| Tumor Volume | 1.00 (0.99-1.02) | | 0.57 | 1.01 (0.99-1.03) | 0.20 |
| Multifocality |  | |  |  |  |
| Yes | 1.55 (0.87-2.76) | | 0.13 | 7.37 (1.47-36.9) | **0.015** |
| No | Reference | | | Reference | |
| Preoperative KPS |  | |  |  |  |
| ≥ 70 | Reference | | | Reference | |
| < 70 | 1.54 (0.86-2.77) | | 0.14 | 0.80 (0.13-4.94) | 0.81 |
| RANO categories |  | |  |  |  |
| Class 1 (n=6\|5) | 0.98 (0.27-3.56) | | 0.98 | N too small | |
| Class 2 (n=19\|19) | Reference | | | Reference | |
| Class 3 (n=14\|16) | 0.87 (0.35-2.18) | | 0.77 | 1.91 (0.67-5.44) | 0.23 |
| Class 4 (n=54\|1) | 1.54 (0.69-3.40) | | 0.29 | N too small | |
| MGMT status | Used for stratification only | | | | |
| Treatment | Used for stratification only | | | | |
| MGMT Methylated Subgroup | | | | | |
| Age | 1.04 (1.01-1.07) | | **0.019** | 1.01 (0.98-1.04) | 0.68 |
| Tumor Depth | 1.01 (0.99-1.03) | | 0.34 | 1.01 (0.99-1.02) | 0.51 |
| Tumor Volume | 1.00 (0.99-1.01) | | 0.80 | 1.01 (1.00-1.02) | 0.054 |
| Multifocality |  | |  |  |  |
| Yes | 1.47 (0.72-3.00) | | 0.29 | 1.54 (0.84-2.83) | 0.16 |
| No | Reference | | | Reference | |
| Preoperative KPS |  | |  |  |  |
| ≥ 70 | Reference | | | Reference | |
| < 70 | 1.43 (0.75-2.72) | | 0.27 | 1.06 (0.44-2.55) | 0.89 |
| RANO categories |  | |  |  |  |
| Class 1 (n=9\|5) | 0.34 (0.10-1.18) | | 0.088 | 0.80 (0.23-2.87) | 0.74 |
| Class 2 (n=37\|47) | Reference | | | Reference | |
| Class 3 (n=25\|36) | 1.22 (0.61-2.44) | | 0.57 | 1.63 (0.98-2.72) | 0.059 |
| Class 4 (n=31\|6) | 2.05 (0.95-4.42) | | 0.068 | 1.21 (0.40-3.70) | 0.73 |
| Treatment | Used for stratification only | | | | |

Table S3: Center-specific results of the multivariable Cox proportional hazards models. HR: hazard ratio; CI: confidence interval; KPS: Karnofsky performance status; MGMT: O_6_-methylguanine DNA-methyltransferase.


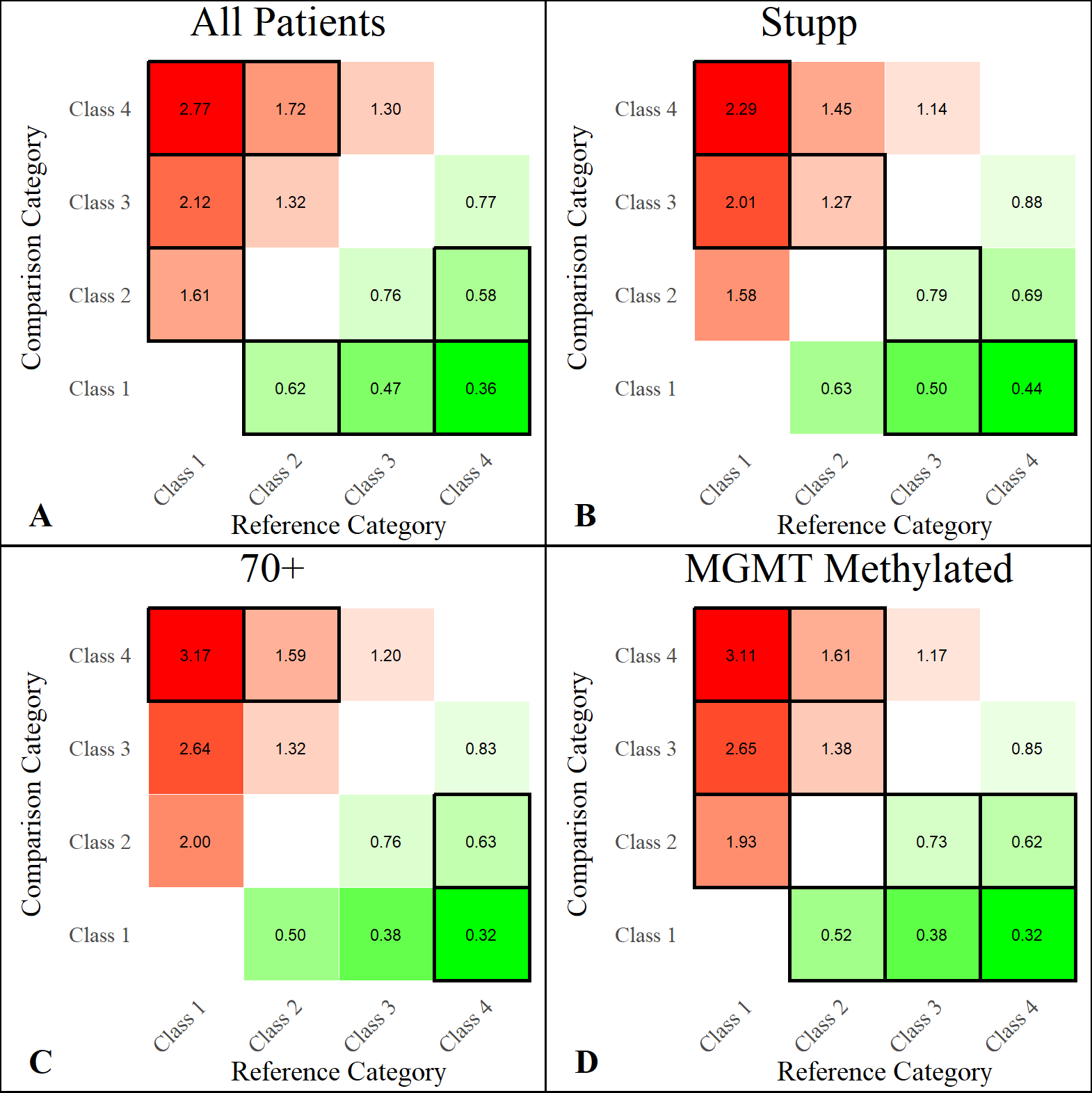


Figure S1. Tile plots showing pairwise comparison of the RANO categories in all patients (A), patients started on treatment according to the Stupp protocol (B), elderly patients (C), and patients with MGMT methylated tumors (D). The numbers are hazard ratios retrieved from multivariable Cox models for the respective subgroups. Statistically significant estimates are outlined in black.
